# ‘Bare-bones’ to ‘silver linings’: lessons on integrating a palliative approach to care in long-term care in Western Canada

**DOI:** 10.1186/s12913-021-06606-x

**Published:** 2021-06-28

**Authors:** Denise Cloutier, Kelli I. Stajduhar, Della Roberts, Carren Dujela, Kaitlyn Roland

**Affiliations:** 1 Department of Geography, University of Victoria, 3800 Finnerty Road, Victoria, BC V8P 5C2 Canada; 2 Institute on Aging and Lifelong Health, University of Victoria, 3800 Finnerty Road, Victoria, BC V8P 5C2 Canada; 3 School of Nursing, University of Victoria, 3800 Finnerty Road, Victoria, BC V8P 5C2 Canada; 4 Palliative & End of Life Care, Island Health, 1952 Bay Street, Victoria, BC V8R 1 J8 Canada; 5 School of Public Health and Social Policy, University of Victoria, 3800 Finnerty Road, Victoria, BC V8P 5C2 Canada

**Keywords:** Palliative care, Long-term care, Care team, Quality of life, Life limiting conditions; trajectory

## Abstract

**Background:**

‘Whole-person’ palliative approaches to care (PAC) are important for enhancing the quality of life of residents with life-limiting conditions in long-term care (LTC). This research is part of a larger, four province study, the ‘SALTY (Seniors Adding Life to Years)’ project to address quality of care in later life.

A Quality Improvement (QI) project to integrate a PAC (PAC-QI) in LTC was implemented in Western Canada in four diverse facilities that varied in terms of ownership, leadership models, bed size and geography. Two palliative ‘link nurses’ were hired for 1 day a week at each site over a two-year time frame to facilitate a PAC and support education and training.

This paper evaluates the challenges with embedding the PAC-QI into LTC, from the perspectives of the direct care, or front-line team members. Sixteen focus groups were undertaken with 80 front-line workers who were predominantly RNs/LPNs (*n* = 25), or Health Care Aides (HCAs; *n* = 32). A total of 23 other individuals from the ranks of dieticians, social workers, recreation and rehabilitation therapists and activity coordinators also participated. Each focus group was taped and transcribed and thematically analyzed by research team members to develop and consolidate the findings related to challenges with embedding the PAC.

**Results:**

Thematic analyses revealed that front-line workers are deeply committed to providing high quality PAC, but face challenges related to longstanding conditions in LTC notably, staff shortages, and perceived lack of time for providing compassionate care. The environment is also characterized by diverse views on what a PAC is, and when it should be applied. Our research suggests that integrated, holistic and sustainable PAC depends upon access to adequate resources for education, training for front-line care workers, and supportive leadership.

**Conclusions:**

The urgent need for integrated PAC models in LTC has been accentuated by the current COVID-19 pandemic. Consequently, it is more imperative than ever before to move forwards with such models in order to promote quality of care and quality of life for residents and families, and to support job satisfaction for essential care workers.

Increasing numbers of older adults are living and dying in long term care (LTC) settings, as a result of increased life expectancies [1, 2]. At the same time, residents who enter LTC are recognizably more complex, experiencing higher levels of acuity than in past decades, and staying for shorter periods of time [3, 4].

Although many LTC facilities today lack substantial, formalized palliative programs and services to support residents with life limiting conditions; appreciation for integrated palliative approaches to care (PAC) has been growing, [4–8].

In recent history, models and approaches to palliative care have tended towards two polarities; specialized services focused on the last days and weeks of life, or whole-person palliative models that support holistic care aimed at providing care to individuals appropriate to where they are at on their own unique health and illness trajectory [3, 9, 10]. Distinguishing palliative care as an approach and philosophy in this latter way, rather than merely as a set of services provided in the last days, is an important progression for many clinicians, researchers and care settings [8, 11, 12].

In addition, PAC reflects person-centered, upstream *approaches* to care, and care planning, in relation to disease management, advancement, and eventual death and supports all individuals with life-limiting conditions (e.g., cancer, heart disease, dementia), wherever they are at on their trajectory as noted [6, 12]. Pain and symptom management have always been a cornerstone of PAC, because of earlier emphases on the care of persons living with cancer. Today, in addition to pain and symptom management, a holistic PAC also attends to a wide range of psychological, spiritual, social and emotional needs where possible [12]. These domains are interrelated, dynamic, and evolving when it comes to care planning for those with life limiting conditions [13].

Adopting and integrating a PAC into usual care supports improved decision making within care planning, provides a range of options for compassion and comfort care, and gives residents greater choice regarding the place of death [14, 15]. After death has occurred, assistance with grief and bereavement for families, and care team members is also important [12].

Currently, the provision of PAC in LTC can be said to be made more challenging due to longstanding circumstances and conditions in this sector. Some of the broadest, and most deep-seated challenges that have been identified are linked to: resources, leadership, organizational support, team climate and coworker support, the training environment, feedback systems, and responsiveness to change [16]. Complicated dynamics between family members and residents can also influence access to PAC, and outcomes [17].

In facility environments, the implementation of a PAC is complicated by the fact that there is often no standardized agreement, definition, or clear policy guidance on what a PAC is, i.e., how it serves to promote quality of life and dignity, or when it should be applied [18, 19]. Successful integration of PAC therefore, requires a combination of factors and actions such as: a workforce who have substantial existing education, experience, skill and confidence in holistic PAC, and an abiding interest in caring for dying individuals, and ongoing access to education, training, and the support of leadership around PAC. Investments that support relationship-building, and conversations and communication regarding death and dying, wishes, values, and preferences in the day to day encounters and interactions with residents and family members are also critically important [12, 20–22].

Another area of challenge linked to integrating PAC in LTC relates to who is considered to be part of the care team, or stated differently, the degree to which the voices of all care team members (registered nurses (RNs), licensed practical nurses (LPNs) and health care aides (HCAs) are heard and acted upon in regards to care planning and decision-making [23, 24]. Specifically, while HCAs and LPNs arguably have the most frequent interactions with residents in relation to daily care, and opportunities for conversations about what matters most to residents, their perspectives may not always be included in care conferences, discussions with physicians, and other clinicians when it comes to resident goals of care [25, 26].

Related to team work in LTC, Sawatzky et al., [27] have highlighted the importance of scope of practice questions in the delivery of PAC. While it is important that care teams be flexible and nimble in addressing the diverse and dynamic needs of residents, different skill sets and competencies are regularly required. Thus, while the flexibility of care team members to jump in when needed is important, quality of care for residents also depends upon the skill and effectiveness of care team members all working individually to their fullest scope of practice, and also working effectively as a team, to promote well-being, quality of life, and dignity for residents [12, 28].

Compared to other OECD countries, COVID-19 mortality rates in Canada reveal a much greater proportion of deaths occurring in LTC, with approximately 81% of deaths taking place in LTC facilities and retirement homes [29]. And, with accelerated rates of death among those aged 85+ living in LTC, interest in PAC has been heightened. Forced restrictions on family engagement upon entry into LTC, and at the end of life as a way of reducing the spread of the virus, have been hard on all of those involved, but particularly on residents, family, and care team members [30].

## Study context and aims

This qualitative research study is part of a larger, four province project called, “Seniors - Adding Life to Years” (SALTY). SALTY is a collaboration between researchers, care providers, care administrators, policy makers, patient advocates, knowledge users, and most importantly, older adults and their families, all working together to enhance quality of life, and improve the quality of care in residential LTC settings. Our paper focuses on understanding the impacts of a palliative approach to a care-quality improvement initiative (PAC-QI) on members of the direct care workforce, most notably RNs/LPNs and HCAs. The PAC-QI initiative was developed by a clinical team of palliative care specialists, in consultation with an advisory committee of LTC specialists, to support the quality of life, and quality of care provided to residents with life limiting conditions living in LTC. Knowledge from PAC models developed in other local, national, and international jurisdictions guided the development of the PAC-QI.

Four diverse LTC sites from a health region in Western Canada participated in the PAC-QI project. These sites encompassed private and public ownership; large and small bed sizes; and urban and rural locations. In addition, they were each unique in terms of their models of leadership and care team composition (i.e., numbers of admitting physicians, nurse practitioners, RNs, LPNs, HCAs, educators and allied health professionals). Two part-time palliative specialist ‘Link’ nurses were hired by the health region and not by the facility in which the project was taking place, as part of the PAC-QI project to spend 1 day a week, or two-half days/week at each site for the two-year duration of the project. These ‘Link nurses’ worked with palliative care physicians and site leadership to introduce a suite of tools, and support education and training on their use in regards to PAC. A fuller discussion of the tools and their impacts is the focus of another paper (Stajduhar, Cloutier, Roberts, Dujela, Roland: Why context matters: the muddy reality of implementing a palliative approach to care in long-term care, In progress).

Many of the tools embedded in the PAC-QI were deemed to be highly valuable to participants. These included: an Early Identification Tool, a Conversation Guide, and, the introduction of palliative rounds to introduce and support the use of the PAC tools, and support care planning and care decisions. The first two tools were effective in providing care team members with references that included a checklist of indicators related to identifying an advanced disease trajectory, and scripts, role-plays, and guides to support having conversations with residents and families about expectations, wishes and values around death and dying.

Distinct from the PAC-QI project, this paper addresses the question, “What were the main challenges with embedding a palliative approach to care (PAC) at your site from your perspective?”

## Methods

### Design

Prior to data collection, ethics approval was received from the harmonized human ethics review board (joint university and health authority board), and from a separate ethics review carried out by the health region in which the project was set. This qualitative study draws on data primarily from focus group interviews with direct care team members (RNs, LPNs and HCAs) undertaken between June 2017 and December 2018. Focus groups were chosen as the main method of data collection to allow for the diverse perspectives of RNs/LPNs and HCAs to be represented. A second reason for the focus group methodology was to gather a wide range of information on a basis that disrupted the work of direct care team members the least. Direct care staff were divided into two groups, non-licensed workers (HCAs), and professional staff (RNs/LPNs) to foster putting more ‘alike’ groups together in order to diminish any sense of power-over relationships which would potentially have limited all voices being heard. Background survey data were also collected from direct care team members between June–September – 2017. Table 1 data to describe characteristics of the direct care workforce is drawn from these surveys.

**Table 1:** Characteristics of Focus Group Participants (Direct Care Workers)

Variable	Health Care Aide or similar (***n*** = 28)^**a**^	Professional (RN/LPN) or similar (***n*** = 42)
% Female	89.2	95.2
Age (mean)	42.8	46.5
% Canadian born	74.3	73.7
Hours per week worked	34.8	30.7
Years at facility	6.8	5.2
Years in residential care	13.2	8.4
Education (% Bachelor + Masters)	27	69
% Part-time staff	18.9	28.6
% Received formal PAC training	60	71.1

^a^Note: The number of participants noted in this table is different from the numbers highlighted for the focus groups because more individuals were invited to complete background surveys than could ultimately participate in the focus groups

### Participant recruitment and procedures

Both facility administration (management/leadership), and the Link nurses helped with recruitment for the focus groups, to understand the impacts of the PAC-QI on direct care team members. These focus groups were advertised via posters in the nursing stations and staff rooms at each site. Focus groups were then scheduled and held during lunch breaks, and pizza was provided as a way of thanking team members for their time and participation. A total of 16 focus groups (two at each of the four sites; and at two time intervals were undertaken; 2 × 4 × 2 = 16). Data collection was timed so that each of the four facilities had at least 6 months of experience with the PAC-QI project before any focus groups or interviews were conducted.

Among participants, the regulated professional staff group consisted of 10 RNs and 15 LPNs and other professional staff such as social workers, occupational therapists, recreation therapists, and dieticians (*n* = 9). The non-licensed group included 32 HCAs; and non-licensed recreation and rehabilitation assistants. (*n* = 13) In the transcripts that were developed, it must be noted that while the voice of the participant was distinguishable in the transcript, and the group they were in, their role was not identifiable. In presenting the findings, extracted data from the focus groups are referred to by the number assigned to the person speaking, followed by a pseudonym for the facility, followed by the designation of whether the individual speaking was from the RNs/LPNs focus group, or the HCA focus group. (e.g., [4, Townside, RNs/LPNs]).

Before beginning the focus groups, participants answered background questions related to their education; length of time spent working in their facility; and their current role in the LTC setting where they were interviewed. Focus groups then moved to framing definitions for what was meant by ‘life-limiting conditions,’ and a ‘palliative approach.’ As defined by the PAC-QI initiative, life-limiting conditions referred to chronic conditions expected to limit how long a person has to live, including dementia, and lung, kidney, heart disease, and cancer. A palliative approach was defined as ‘an approach to care focused on improving the quality of life of persons with life-limiting conditions and their families.’ Next, the focus groups addressed questions about how the project was experienced by direct care team members, and what some of the challenges with its implementation were. Focus groups ranged in length from about 28 to 64 min.

Table 1 provides a breakdown of these data by group in terms of HCAs versus RNs/LPNs.

The workforce at each of the four sites was predominantly female (89% for HCAs and 95% for RNs/LPNs), and around three-quarters were Canadian born for both HCAs and RNs/LPNs. The mean age of HCAs was about 43 years old versus 46 for RNs/LPNs. Team members had an average of about 11 years of experience working in residential LTC, but HCAs had 13 years; while RNs/LPNs had 8 years. Each group had less years in their particular facility. Formal palliative training of some kind was reported by 60% of HCAs versus 71% of RNs/LPNs.

### Data analysis and interpretation

All focus groups were taped and transcribed. Transcripts were entered into NVivo 10 qualitative software for thematic analysis [31–33]. An initial coding dictionary was developed by two team members who read the transcripts from the first four focus groups that were conducted. These four transcripts initially represented two facilities, and the corresponding transcripts for primarily RNs/LPNs participants, and primarily HCA participants. The two team members worked independently initially to categorize and summarize their individual findings. Next, they worked together to develop an initial coding structure that was then tested and fleshed out by the broader team, who read the same initial four transcripts [32, 33]. After consensus on the final coding scheme was reached, all data were coded in NVivo by a post-doctoral researcher member of the team.

Once coding had been completed, team members then met on a regular bi-monthly basis to review emerging findings and summaries, and request additional data extractions, in order to build a fuller understanding of the data, and to establish an understanding of the priority themes and goals for building consensus, validating findings, and developing papers for publication [31–33]. Data nodes that were examined for this paper were broadly related to the themes of context, and barriers and successes with respect to embedding the PAC from the perspectives of direct care team members [31–34].

### Findings

This research aimed to understand the impacts of a PAC-QI project on direct care team members (primarily HCAs, LPNs, and RNs). Generally speaking, care team members exposed to the PAC-QI project, expressed their strong appreciation for the education and training, tools and support provided by the Link nurses throughout the project. Challenges that emerged with integrating a PAC are captured under three key themes with related subthemes. The main themes are: (1) longstanding challenges in the LTC sector that were present before the PAC-QI project, e.g., chronic staff shortages and lack of time for care provision; (2) a diversity of viewpoints about what a PAC is, and when it should be applied; and (3) differential access to education, training and support among care team members. It is important to point out that these three main thematic areas, and many of the identified subthemes are often overlapping rather than distinct.

### Longstanding challenges within the long-term care landscape

#### An environment under stress

First and foremost, it is critical to acknowledge direct care workers’ deep commitment, and sense of feeling honoured, to be able to care for, and companion older adults with life-limiting illness throughout their LTC journey, and especially at the end of life. One participant in the RNs/LPNs focus group said: “…for me, palliative care, that’s always been the rewarding part of it, is being able to be there for them, where families aren’t often able to be there in their dying moments, and you know I mean it’s a privilege…”. [4, Eaglebluff, RNs/LPNs]

At the same time, many RNs/LPNs and HCAs said their workplace was a challenging environment. Participants from the RNs/LPNs group indicated: “we spend all of our time putting out fires,” [1, Meadowview, RNs/LPNs] while an HCA group member noted their environment is in a constant state of “upheaval.” [1, Townside, HCAs]. Another HCA added “I work on a very busy floor and sometimes short staffed. Management seems not to care about heavy workloads I have.” [3, Meadowview, HCA]

One key reason why LTC workplaces are challenging today has to do with the increased acuity of residents, and their shorter lengths of stay on average. Today, residents arrive in LTC with greater frailty and higher complexity than in decades past. Some participants described their residents as exhibiting reduced mental health, and difficult behaviours such as aggression and agitation. A number of care team members commented that residents are ‘closer to death’ when they arrive, and with shorter stays, it becomes more difficult for care team members to build relationships, and get to know them ‘as people.’

#### Time and staffing

On the human resource side of things, the most challenging aspects of integrating a PAC in LTC for participants were arguably related to chronic staff shortages, and consequent perceptions of having no time to provide the care they want to, and that they feel residents need. These realities underscore that the PAC-QI project was an attempt to integrate a PAC in a system that had already been under notable stress for several decades.

When asked what affects your ability to provide the care you want to, a RNs/LPNs focus group member said:

…for most of us, uh, time constraints are, are a huge, huge, huge piece. Um, certainly from a nursing perspective there are seventy-two residents, and you have to divide your time between, between all seventy-two [1, Townside, RNs/LPNs]

Similarly, an HCA focus group participant highlighted the tensions of juggling care for multiple residents in a context of staff and time pressures:

….one of the residents was in a palliative state to the point where they’re bedridden and they can’t move around, and if you don’t have the proper staff and you’re out in the dining room trying to feed everyone lunch, then you don’t have the time to be in the room. Well, what if they don’t have any family members at that time? Now you’re short staffed, you’re trying to run around to care for all the people that are up and about and moving around and keeping an eye on them, while you’re almost feeling like that the person that is laying for most of the night there is being neglected [2, Eaglebluff, HCAs]

#### Resident-staff ratios

Over the last decade, ratios of the number of residents to number of staff have been going up according to many participants. One LPN from the RNs/LPNs focus group said, “The LPNs workload doubled last year. I have 40 residents. It makes it very frustrating, and hard and emotional. Because there’s a reason we’re here, and [why] we’re still here, because a lot of the staff have gone… we are exhausted, overwhelmed. Because the expectations are … they are just off the charts.” [3, Eaglebluff, RNs/LPNs]. The sense of overwhelm among care team members has several causes, but for many participants came down to having too few staff to do the work that is required. Many expressed the view that they work short-handed all the time; with care team members not being replaced if someone calls in sick, or goes on holiday; whether they work a day shift or a night shift; and whether they are full-time, or part of the casual workforce.

Team members noted that the nature of the work they do for residents is also influenced by time and staff. In recent years, additional pressures related to increased paperwork and chart audits have been added to, or amplified in workloads. These take time away from resident care. Several members of a RNs/LPNs focus group vocalized a view that they feel that “they barely have any time to read care plans anymore” much less to ask residents what they want [4, Meadowview HCAs],” summing up their perception of the crunches wrought by lack of time, and lack of staff by stating, “Again - time and too many patients, LPNs have on average 28 patients. Hard to spend time with 1 or 2 palliative patients (or more) when you have so many others and so much work to do…” [1, Douglascliff, RNs/LPNs]

#### Nature and continuity of care

A RN/LPN participant offered the following: “When we have no time, we have to focus on physical care only. We do the basics—do assessments and give medications.” [4, Meadowview, RNs/LPNs] This excerpt highlights how trade-offs are weighed and decisions made when time is short. In addition, tasks like pain management are critical, but can impact other domains that are part of the whole-person philosophy of PAC such as meeting some of the emotional, social and psychological needs of residents.

In the current milieu, a lack of time and staffing also influences continuity of care especially at shift changes according to a RNs/LPNs participant:

“…used to be you would look forward to coming to work. Now, the workload is much more, and I don’t know if there’s quite that feeling anymore. The other thing about time, um, and looking back, there used to be report time built into rotations for the 24-hour caregiving staff, nursing and care aides. That’s gone. There’s no overlap of shifts, even for fifteen minutes, to hand over well, so people do it on their own time, and it’s rushed.” [2, Townside, RNs/LPNs]

#### Heightened distress in not being there

These quotations also signify how a lack of time and staff can translate into feelings of distress among care team members related to their sense of not being there for residents especially in their dying days. A HCA participant summed up her sense that: “The hardest part of my job is end of life care. [2, Douglascliff, HCAs]

A RNs/LPNs focus group participant echoed similar sentiments:

…I don’t even have time to spend at an actively dying resident’s bedside. I have time to get orders, I have time to administer medication and pop in to see that they’re comfortable, and that’s it. There’s very little personal TLC there. It’s, it’s kind of ‘bare bones.’ [5, Eaglebluff, RNs/LPNs]

### Diverse and shifting views about palliative approaches to care (PAC)

When the PAC-QI initiative was launched with the support of the palliative Link nurses at each site, the initial focus was on caring for residents in their last 6–12 months of life. As the project evolved, this focus broadened to emphasize who could benefit from a whole-person PAC, whether at entry into LTC, or anywhere else along their illness trajectory. In this way, the PAC-QI project is distinguishable from approaches that emphasize the care provided in the last days and weeks of life. At the same time, it was apparent that a consistent top-down, and bottom-up vision for PAC was not always evident in the four facilities.

A RNs/LPNs focus group participant said:

…attitudes are important, you know, there was one nurse in particular who had the palliative approach so firmly entrenched as part of her psyche, but her understanding of the palliative approach was different than mine. Her idea of a palliative approach was final days, and really ramping up medications… [1, Douglascliff, RNs/LPNs]

In the broadest possible view, a member of a HCA focus group indicated: “I would love to think my approach to care with everybody in this building is a PAC, but many things get in the way of being able to do that.” [1, Douglascliff, HCAs].

#### Receptiveness to a palliative approach to care (PAC)

How a PAC is interpreted and enacted by direct care team members, and positioned by leadership, is part of the challenge with establishing a unified vision that can be mobilized and embedded. This is partly illustrated in the following statement by a RNs/LPNs focus group participant who said: “[The site] has pre-ordained palliative orders…it’s very nondescript. It’s not tailor made. It doesn’t deal with the resident at all… They will just carte blanche put a set of orders in place.” [2, Meadowview, RNs/LPNs]. In this case, leadership is recognized as being supportive of PAC given the existence of palliative orders, but the way the PAC is interpreted and acted upon by team members can be variable. This suggests there can be tensions between orders, and role expectations for care team members, that is, the extent to which all team members, feel empowered to work effectively with palliative orders, and work to their full scope of practice and still provide the kind of personalized care needed to promote quality of life for residents.

An RN at a site that faced more challenges with embedding the PAC highlighted a complex range of issues related to embedding it;

“….graduating nurses with not enough bedside experience; a 1 week hospice course would be helpful, but not funded. The pilot project [QI] has highlighted that the workplace is not open to a palliative approach. In addition, health care practitioners personal beliefs negatively impact care provision. For example, breakthrough meds may not be given because they believe it will kill the residents. New graduates are poorly educated, for example, [they] can take blood pressure but don’t know what it means. Physicians are not attending patients. Palliation is not done well and having RNs on the same page would provide clinical leadership. LPNs are not receptive to the program. [3, Meadowview, RNs/LPNs]

A HCA participant revealed how the QI had spurred them to reflect more deeply on their own care practices:

Since becoming aware of this palliative project that endeavours to educate and support health care workers in their approach to those with life limiting conditions, I have had a chance to re-evaluate my own personal attitude and actions. As an enlightened society, we need to put a true value on the dignity and understanding of the effects of our approaches on those individuals. [4, Eaglebluff, HCAs]

#### Relationship-building and person centered care

Other subthemes that emerged in relation to a common vision for PAC emphasized the fact that the application of PAC may benefit from having even more staff per resident than might be the case for usual care. High quality PAC requires a committed focus on building-relationships with residents, according to some participants; rather than only focusing on emergent needs. Some said that ‘softer,’ ‘slower,’ more ‘mindful care’ is important. Slowing it down also means being able to pay attention to the little things in people’s lives like having a conversation, even if short, each day with someone to see how they are doing. Overall, enacting PAC requires a common vision, and an adequate level of care staff to deliver that care.

### Implications of differential access to education, training and support

The majority of care team members who had been exposed to the PAC-QI (the Link nurses, tools and education, training and support opportunities), had strong praise for the project as having several ‘silver linings’. Most notably, the project supported identifying those on a dying trajectory sooner, and supported staff with skills and language to have conversations with residents and families about death and dying at the earliest stages. However, after talking to care team members who participated in the PAC-QI, it was also apparent that it was integrated to varying degrees at each of the four sites, which is understandable in relation to their differing contexts in terms of size, care team composition, philosophy and leadership.

#### Voices of direct care team members

Sometimes there was limited awareness that the PAC had been introduced at certain sites. Further, representatives of the HCA workforce who provide the most direct hands-on care to residents, indicated having limited ability to apply PAC at some sites, or to receive palliative education more generally, due to high ratios of residents to workers as already noted, but also because they could only get access to PAC training if their position was backfilled. To this point, one HCA focus group member said, “It would be difficult for us [to receive training] because they want us to be there, but then they don’t make sure that we have the appropriate time to be there, or that our areas are covered, so that we can be there without leaving the people we’re taking care of.” [1, Meadowview, HCAs] When asked if they get *enough* education in general, or specific to PAC, many HCA participants gave a flat “No,” while other HCAs conveyed a view that if they wanted to take a course on palliative care, they would likely have to do it on their own time, possibly using their own money as well. These excerpts highlight the tensions that exist at sites between the ability to benefit from PAC training, but also whether PAC is adopted as a whole-person approach to care, or a set of services. Although informal support might be available for some, this is not always the case as shared by this HCA: “At our facility we are afraid to ask for help from our RNs, as we are disciplined for not knowing something.” [3, Eaglebluff, HCAs].

#### Shifting the long-term care (LTC) culture to support palliative approaches to care (PAC)

Many participants also spoke about barriers to changing practice, reminding researchers that even with improved access to education and tools, making changes to usual care routines is difficult. One participant in the RNs/LPNs focus group offered this reflection about caring for dying individuals, which reflects changing practice, and also the imperfect science of being able to definitively identify people on a dying trajectory:

“One man who came in, died two or three weeks later. He came in palliative, but we were getting him up, getting him dressed. He didn’t want to eat dinner. Why were we taking him to dinner? Because that is what we do. We don’t know, so we just keep doing it…. You give him a meal and he won’t eat a thing, and to me that’s a sign somebody’s declining. He’s skinny, he wants to sleep. You got him up, but he wanted to go back to bed. Now, we did that for two weeks. And, I’m thinking, why from the start didn’t we observe that, and maybe for a few days had the routine, and then go ‘“No, I think this man is dying.” [2, Meadowview, HCAs].

Even though resident acuity has increased in recent years, this man’s illness trajectory was very steep, and declining from the moment he entered LTC. His experience underscores the importance of promoting education and training for all care team members regarding palliative approaches to care from the early identification tool, and relatedly, to support having conversations about death and dying, as soon as possible with residents, to enhance quality of care and quality of life from their time of entry. Early conversations between residents, families, and team members help to ensure care planning aligns with the wishes, hopes and preferences of residents at all points along the illness trajectory.

#### Dealing with grief

One area that was highlighted by care team members as being a potential gap in the training available within the PAC-QI was related to dealing with the loss of residents. Care team member participants highlighted the fact that to their knowledge there were no educational resources available to them to help them to deal with their own grief. A member of the RNs/LPNs focus group said: “We don’t *in*tend to get really close with certain people, but we tend to, …it’s a human nature thing to do so, [therefore] some deaths might be harder for us than others.” [4, Douglascliff, HCAs].

Another HCA focus group participant expressed their gratitude for being encouraged to work with their own musical skills as a care team member to play for residents in their growing frailty, but emphasized this point that management needs to understand, and help workers with their grief.

So, like I said earlier, coming into the building and then sort of, everyone got excited when learning I was a musician. Like, oh great, you can go and see these people because they will benefit so much from the music. And, I did end up playing a lot of music at the bedside of the people who had only a few days left, and it was amazing, and I really appreciated the experience, except that I was so overwhelmed by the amount of people dying, and I’d never been around that ever before in my life, so I didn’t feel that I had the resources in me to really handle that properly.…there was not even sort of like a printout or something anywhere that said how to cope, or these are the 10 steps for dealing with the loss of, you know, people that you care about. [2, Meadowview, HCAs]

In summary, the need for ongoing education, training and leadership support across the range of team members, and over time is important. Facilities need to recognize, as well as work with, and build upon the skillsets and experiences of their team members.

## Discussion

To our knowledge, this study is one of the first in a Canadian context to address a critical gap -- our need to better understand the barriers and opportunities related to integrating palliative approaches to care in a LTC context. If nothing else, the current global COVID-19 pandemic has shone a penetrating, and heart-rending light on LTC as the place in which higher numbers of older persons increasingly live and die [3, 10, 18]. As a place of care and comfort, the LTC environment is incredibly complex. There are multiple factors at play in a dynamic, and ever-changing environment. In this paper, we have evaluated a range of challenges related to embedding a palliative approach to care in LTC including longstanding issues in LTC before the advent of the PAC, alongside increasing client acuity, and renewed and amplified demands on care team members in the last decade [7].

In our study, we evaluated the main intentions of the PAC-QI itself which were to foster more holistic, or whole person PAC, as something that individuals with life limiting conditions could benefit from upon entry into LTC. The main intentions of this paper were to understand the experiences of direct care workers, primarily RNs/LPNs, and HCAs with the implementation of the PAC-QI project in four facility settings.

In future, growing numbers of older adults will live in LTC as a function of increasing population longevity. Their stays will tend to be shorter since they are entering LTC with increased acuity and complexity. It is paradoxical that while there are more people living and dying in LTC than before, the system has never been more fragmented and fraught with challenges. Arguably, the greatest challenge to the successful implementation and ongoing success of the PAC-QI, was that it was predicated on an existing system already besieged by chronic underfunding, ongoing devaluing of staff, and their credentials and experience, and overall human resource shortages [35, 36]. Despite their deep commitment to providing the best whole-person care that they can, direct care team members underscored the challenging nature of their work environment in terms of the pressures of too little time and too few staff. They foretold how the workforce is ‘too slim on the ground,’ and how they are going flat out all the time, working shorthanded more often than not, and constantly feeling that the care they are providing is ‘bare bones,’ or less than they would like to provide [37, 38].

### Vision

This research also highlighted the importance of a consistent vision for PAC within facilities, one that is recognized, understood and supported at all levels; from management, on down to direct care workers [8, 13]. Increasingly, it seems equally important for the overarching philosophy of care in LTC, to be directly aligned with the philosophy of PAC, given that with their high complexity and acuity, the majority of residents can be considered to be on a dying trajectory from the time of entry into LTC. Recognizing where individuals are at on their trajectory, helps in care planning that prioritizes quality of life concerns in the development of practices and protocols [12]. Put another way, focusing attention on both quality of care and quality of life in LTC, means committing to the fullest extent possible to the integration of a PAC based on an extensive and accurate assessment of each resident initially, and at regular intervals throughout their stay in LTC. These assessments also need to consider each resident’s needs, hopes, and wishes.

While the PAC-QI project supported staff to attend education days, and Link nurses supported PAC education and training for team members, our findings highlighted that access to education and training was not equitable for all direct care team members. In particular, health care aides who provide the most hands-on-care, were not always able to benefit from such opportunities unless their positions were backfilled, or they worked day as opposed to night shifts, or they took on the training on their own time, and sometimes using their own resources.

Our findings also suggest that the adoption or embedding of a more integrated PAC has a greater chance of success if all care team members have access to some level of training that focuses on early identification of the dying trajectory; preparatory conversations about death and dying; and the management of expectations for residents, families and team members towards the end of life [12, 18]. Care team members build competencies working as part of a highly trained team each working to their full scope of practice. Past experiences also build individual capacities to provide effective PAC. This acquired knowledge should be valued and supported by leadership when it comes to having some of their own needs met in relation to dealing with stress and burnout, and their grief over the loss of residents [39]. Ultimately, resources to enhance and improve access to education and training related to all aspects of PAC will be paramount for the success of these models. Education must be recognized as an ongoing priority and resource requirement [40, 41].

We have noted that ‘whole-person care’ means that care team members must be able to assess quality of life among residents and attend to their spiritual, emotional and mental health needs [3]. In our study, one example of this was the direct care worker who was encouraged and supported to play music at the bedside of residents who were actively dying. Attending to the physical needs of residents also remains crucial especially since pain can still be under recognized and under-managed [42, 43].

The PAC-QI project evaluated here may be described as a hybrid model because it recognized the need to first build upon the existing internal capacities of organizations, and second, to infuse new resources into the system [10]. New resources entered the system for the duration of the PAC-QI in the form of the palliative Link nurses who helped with education around what a PAC approach was, and who also supported education and training about tools such as the early identification of the dying trajectory tool for direct care team members as already highlighted. The majority of focus group participants who had been exposed to the PAC-QI, were appreciative of the additional knowledge and skills they gained. Taken together, integrated PAC serves to develop a more effective and confident care team [42–46], and enhances the likelihood of providing more appropriate, tailored and compassionate care for residents and families [10, 47].

It could be argued that the PAC-QI was too short to promote sustainable system change since after the funding stopped, and the Link nurses were no longer visiting each site, opportunities for ongoing education, training and support were jeopardized at some sites more than others. In this manner, the PAC-QI project highlights the need for adequate and ongoing funding and resources to sustain a PAC. Similarly, to an extent, the PAC-QI initiatives raise questions about the limits to creative team work and leadership support and flexibility without an infusion of new and ongoing resources. Without such commitments, it is hard to imagine any of the pressure points within the current system being alleviated effectively.

### Building solutions

In their Mission Statement, the Quality End-of-Life Care Coalition of Canada recognizes that all Canadians have the right to die with dignity, free of pain in a setting of their choosing, and surrounded by loved ones. ([39] , p.1) [12]. Canada currently ranks a dismal 18th in the world in regards to the provision of palliative (Stajduhar, Cloutier, Roberts, Dujela, Roland: Why context matters: the muddy reality of implementing a palliative approach to care in long-term care, In progress) care. [10]. Consequently, the Quality End-of-Life Care Coalition, and the Canadian Hospice Palliative Care Association are regarded as beacons of hope with respect to their blueprints for action, and their commitment to continue to push for the achievement of meaningful, sustainable and integrated PAC to support individuals at end of life [10].

At the organizational level, Kaasalainen et al. [10] calls for multi-faceted capacity building approaches to change culture and change practice in relation to PAC in LTC. Creative solutions include working with champions, finding external mentors, working with expert consultative nurses like the palliative link nurses in this project, and so forth. Having community outreach nurses assist with engaging in reflective debriefing exercises, and supportive ‘comfort care’ rounds, and palliative care rounds are other ideas that have been specifically cited as beneficial for PAC; and were some of the strategies employed here [6, 47, 48]. In other research, Kaasalainen et al. [49] reported that having personal support workers like health care aides shadow hospice workers has been effective in building their capacity to promote whole-person PAC in LTC.

### Limitations

Several limitations to this research should be noted. First, we were unable to incorporate the views of residents and families in this analysis even though we acknowledge their fundamental place as integral members of the full care team in regards to PAC, and to care planning. Moreover, when their perspectives are included, there is a greater likelihood of improved outcomes that will meet resident and family hopes and wishes for a good life, and good death while living in LTC [23, 50]. A second limitation of this research is that the PAC-QI was implemented in four diverse facilities (in terms of size of beds, ownership, location, management type, resident mix, interdisciplinary care team nature and mix, and geography) and at slightly different time points which is characteristic of research carried out in ‘real-time’ and ‘real-world’ laboratories, but which prevents easy pre- and post-comparisons of the impacts and lessons learned. Related to this point, in western Canada, and in other jurisdictions, some research evidence reports that the type of ownership in a facility has a substantial influence on the care provided [42, 51]. In this paper, we did not distinguish how the type of facility may have influenced the PAC-QI integration and the extent of the challenges. Future analyses should consider comparisons of the perspectives of direct care workers relative to the different type of facilities in which they work teasing out the effects of private/public ownership for example, and urban/rural differences as well.

### Future directions

In the literature, a substantial body of research builds a direct link between quality of care or improved resident outcomes, and level of staffing, noting that more staff also have a positive impact on care team members themselves in terms of increasing job satisfaction, lowering rates of burnout, and reducing staff turnover [52–54]. At the same time, although a large research base addresses the importance of educational initiatives, support for multidisciplinary teams; and the need for teams to be able to adapt to change [5]; more studies are needed on how to grow and support the workforce to address many of the challenges that currently exist. Additional studies should also be undertaken that explore the characteristics and qualities of LTC organizations that are successful in embedding effective PAC, or the optimum composition and skillsets of team members to support effective, holistic PAC.

On a related point, alongside increasing the size of the workforce in LTC and the ratio of staff to residents, there is an urgent need for reforms and attention being given to improving the wages, benefits and working conditions for direct care team members in LTC. This is essential to both support the current workforce, and draw students and experts to these professional, and non-licensed jobs and workplaces, to offset losses that are occurring due to injury, stress, burnout, and poor remuneration. Improved roles and support for family members, and volunteers can also not be understated as they form a critical part of the care team workforce who often have limited voices in care planning and decision making.

Finally, it would seem that there is a need for broader organizational (and societal) dialogue on death and dying. Cable-Williams and Wilson [2] argue that a strong barrier to PAC integration is the fact that there is insufficient recognition of the dying process as being intertwined with, rather than separate from, the living process. They argue that too much time is lost in LTC through death-denying belief systems that avoid *any* consideration of death as an inevitable consequence of living. Sadly, such perspectives tend to withhold care that would better support the whole-person, which is so critical to quality of care and quality of life for residents and families. All of this evidence points to a critical need to move full speed ahead to develop, resource, and enhance integrated palliative approaches to care in facility-based LTC settings [5, 7, 10, 55].

## Conclusion

In most countries in the Global North, the number of older adults requiring LTC services to meet their daily needs will continue to grow. At the same time, the COVID-19 pandemic has highlighted how important, and critical the work of RNs, LPNs, HCAs and other care team members are to championing the care that older adults with life-limiting conditions receive on a daily basis. Our findings underscore the important and critical need to integrate and embed palliative approaches to care in intentional, whole-person ways to support the care and quality of life of individuals from the time they enter LTC to their last days.

Going forwards, immediate efforts should be made to ensure holistic, palliative approaches to care become the standard for usual care in these settings. Strategic investments in staff and training are minimum requirements to support quality of life for residents and workers. In a perfect world, there would be adequate fiscal resources and financial rewards to support addressing human resource shortages, and expanding care teams, and ensuring access to effective and ongoing education and training. There is a desperate need for reforms and incentives to ensure a strong and steady supply of trained professional, and non-licensed workers are drawn to this field of caring. Supportive leadership though not the focus of the research highlighted here, is essential to build and support a strong and common vision, and creative solutions to the enactment and embedding of PAC. Deeply concerning is the potential scenario of nothing changing, and of even more limited resources being infused in LTC than in the past, as governments strain to adapt and evolve in a post-COVID world. This means that it will take all existing human and financial resources, as well as all of our ingenuity and creativity to mobilize the current skills and expertise of care team members, residents, families and volunteers to promote the quality of life of residents. It is too be hoped that resources will come soon, and that the lack of resources meantime, will help to catalyze a re-orientation of care towards whole-person integrated PAC in LTC. Being able to capitalize on care team member strengths in different ways, and with better outcomes, while supporting them more fully in the work they do must be pursued vigorously and doggedly. Integrated PAC needs to be recognized as ‘essential’ rather than merely desirable, so that we can protect and care for residents and families, as well as our essential workers.

## Data Availability

Metadata (de-identified) is available through written request to the corresponding author.
